# Tobacco—Its Effects upon the General Health, and Its Influence upon the Teeth

**Published:** 1860-10

**Authors:** 


AMERICAN JOURNAL

OF

DENTAL SCIENCE.

Vol. XI. NEW SERIES.—OCTOBER, 1860. No. 4.

### ORIGINAL COMMUNICATIONS.

### ARTICLE I.

### *Tobacco—its Effects upon the General Health, and its Influence upon the Teeth.*

We have been induced to offer the present article as much to present some interesting facts which have recently appeared in treatises upon the subject, from which we shall borrow largely, as to advance the idea that it is in some cases preservative of the teeth.

We are no apologist for the filthy abuse in the use of the weed, but advocate the position of judging of it, according to its analytical qualities and their effects upon the system, not screening the great social misdemeanor of which it is too often the disgusting evidence. Kingsley is nearly right when he says "man is a smoking animal," for it especially signalises him from all other animals, for about three centuries has it been gaining ground until all civilized nations use it extravagantly, and many of the uncivilized and barbarous do likewise.

It will be useful to our purpose to know by figures how general this use is, so as to draw a conclusion of its vital effects. A writer states that "the tobacco zone girdles the globe." From the equator through fifty degrees of latitude, it grows and is consumed on every continent. All classes, all ages, in all climates, and in some countries both sexes, use tobacco to dispel heat, to resist cold, to soothe to reverie, or to arouse the brain. In Great Britain 17 ounces per head, France 18½, Denmark 70, Belgium 73½, New South Wales 14 lbs., and supposed to be much greater in some of the States of North America. The average for the whole human race of one thousand millions has been reasonably set at seventy ounces per head, or two millions of tons, requiring five and a half million acres of rich lands to produce it, of this the United States alone raised in 1850 two hundred million pounds, at the same time importing eighty-two million pounds from Turkey, Germany and Cuba, giving about three pounds eight ounces per head to our population.

These figures, we think, with the writer, must lead us to conclude that it cannot be so deleterious to the body and mind as the anti-tobacconist would lead us to believe, "or whole nations would have been extinguished under its use." Many mighty ones have used it for centuries and show no aggregated deterioration, individual exceptions being allowed, arising from idiosyncrasy or beastly excess, having no weight in the argument, nor will we ponder to moralize upon the cause inducing man to resort to narcotic stimulants, or any other of his characteristic habits, suffice it that he is beyond all other creatures, the tobacco consumer, our object being to find if this is necessarily injurious, if statistical facts prove that it is destructive to the race, either physically or mentally.

In the first place we start with the proposition that the intellectual condition of certain leading nations is greater than at any previous period, and to all observations making more rapid and powerful advances at this time than at any previous period of their history. We will cite North America, England, France, Germany, Prussia, Belgium, &c., as examples. The records of longevity in these different nations, although in certain localities showing great fluctuation, in the aggregate establish the fact, that the term of life is gradually being prolonged. Instead of any of our most enlightened nations stagnating or deteriorating, they are steadily advancing towards an improved condition of health, proven by the average life of man being greater, at the same time we have the coincidence of an enormously increasing use of tobacco, increase of size, weight, strength and endurance.

All European travelers unite in stating that the armor worn by the ancient as well as more modern warriors, would be much too small for the average men of the present day. The celebrated Black Prince must have been a man whose stature was five feet eight, and weight of about one hundred and forty pounds, less than the generality of men of our times. Of this the writer is confident, having examined the breast-plate, casque and shield critically, as well as many other well known genuine suits, most bearing conclusive evidence of this fact.

The recent wars in different parts of the world show no decrease in man's power of bodily endurance, as may be instanced in the Crimea, as well as in India, where hunger and fatigue were borne by masses of men almost unequalled; where often the only full supply of wants was in tobacco, which proved a sufficient support until more material aid was found. So too in our recent Rocky Mountain expedition, where it proved of the greatest importance and was missed more than the expenditure of any other supply, until a temporary substitute was had in the dried bark of the willow.

We must presume that these points will be conceded—that greater progress has been made coincident with the increasing use of tobacco, by nations who use it most extensively. We therefore have no evidence to prove that it has had a deteriorating result, whilst it may be more reasonable to infer that it may have had a rather good than a bad effect, from the fact of these acknowledged improvements occurring during its greatest use.

Little is hazarded in saying that the great majority of our men of science, art and literature, have indulged in the use of tobacco more or less extensively, many performing their best labors under its enjoyment, and judging from our personal observation, tobacco users live to a ripe old age.

We will now glean from various published documents its chemical characteristics, in the hope of discovering what the true nature of this remarkable plant is. The active matter of the leaves is found to be resident in a volatile alkali *nicotin*, and a volatile oil *nicotianin*, a third is eliminated under great heat, *empyreumatic oil*, these are the active principles involved in inert matters found in most vegetable formations, as starch, gum, albumen, resin, lignin, with the organic acids in varying proportions as is the case in other plants. The inorganic salts predominate in tobacco as those of lime, potassa, ammonia, &c., as do highly nitrogenized substances, making it one of the most exhausting of crops.

Orfila says that *nicotin** has the greatest power to resist decomposition in the decaying tissues of the body, it being found in animals destroyed by it months after their death. It exists in greater or less proportions in all tobacco, but is generally very small in quantity, is a most active poison, odorless, an acrid and burning taste, as found in the plant is not volatile, and in treatment alkaline. The latter tobacco has least in quantity, ranging in the mild from two per cent. to eight in the rankest.

*Nicotianin* as it exists in tobacco, is a mere fact of the laboratory as well as the alkaloid just noticed, except in smoking, for ordinary chewing does not exhibit it to any degree, except that the peculiar smell is thought to be

*It has recently been proposed as an antidote for strychnine.

attributable to the volatile oil of which we are now treating. It constitutes but a very slight proportion, for even by distillation one pound yields but two grains, being one half of one per cent., age supposed to increase this quantity. When inhaled or smelt, it produces sneezing, but taken as medicine in sufficient doses excites vomiting and giddiness, both of these principles are active constituents of tobacco, *nicotin* predominating, and is perhaps much the most important, it being almost impossible to inhale any proportion of its fumes, a single drop destroying any small animal, whilst one grain has been taken of nicotianin with impunity.

It is difficult to imagine the third constituent of tobacco, empyreumatic oil, even by experiment, as separate and distinct from the two first principles, it being an oil like nicotianin, which is comparatively inoperative, but involved with a base of strong narcotic poison, which is destroyed by washing the oil with nitric acid, showing its affinity to the base nicotin, both losing their narcotic, poisonous character by treatment with this acid. Indeed the two former most probably can be found in this oil, which is produced by combustion in partly closed vessels, as the common pipe, and is the coloring material of the German meerschaum clay. The writer from whom we have borrowed so freely says, nicotianin from its feebler action and small amount, is not a very efficient principle in producing the narcotic effects of tobacco, and that the empyreumatic oil consists only of fatty matters holding the alkali in solution. We are forced to believe that the only constituent worthy of much attention, as the essence of the plant, is the organic base, nicotin or nicotiá.

The great question to be solved is, how much of this is absorbed into the system in its uses, and if absorbed, how does it act, for after all we must judge by the effects produced. In smoking, the heat is but sufficient to volatilize a perceptible portion, which is either ejected, or the mouth protects itself by its secretions, and of the supposed two per cent., scarcely a part could be absorbed without the usual symptoms attendant upon its exhibition, and which is only apparent as long as the system has not protected itself against its attack, as in its early use, when all of its injurious effects are no doubt made particularly evident.

The abuse of the most wholesome aliment is attended with injurious consequences, the most appropriate remedy pushed too far becomes a deadly poison at times, and tobacco as vulgarly used may be equally pernicious. But if this is so dreadful a poison its almost universal use should enable us to determine its exact action, and see its results immediately, as in all other similar conditions of slow poisonings, as it is claimed to be.

The opium eater gives indubitable signs of approaching decay at an invariable early age, besides having a peculiar bodily indication of his habit, as well as mental hallucination, so with hatchis, so with intemperate drinking, but tobacco is the regular companion of most of our old men and is not commonly attended with any physical change or appearance except it be in individual cases and in its beastly use. Mankind universally indulge in fruit and other food from which chemistry enables us to distil fearfully rapid poisons, and judging these by the results of their analysis, we would be all slowly but certainly destroying ourselves. Common salt contains the deadliest vapor chlorine, yet from experience who would denounce its use, indeed is it not imperatively demanded by the instinct of all animals? Most fruit contain more or less of prussic acid, a poison quite as active as nicotin, the former more abundantly found in the peach, plum and some nuts, than the latter is in tobacco, yet the imagination of man has not yet attacked these luxuries, as they only come occasionally and are universal indulgences.

No fact is more clear to the true chemist than that the results of his analysis is one thing and that of its chemical appropriation quite another, so that it is neither safe nor reliable to compare the two products. Nature appropriates or rejects the elements brought in contact, by bringing to bear elements and forces quite inscrutable and unknown, often changing instantly the destructive matter into the inert or useful, so that we are ever driven, in such questions, to seek for the known physiological effects from which to form our opinions. Here we take issue with the opposition, and claim that the positive proof of the injurious effects of tobacco is wanting, except in some few exceptional cases, which prove nothing.

The immense indulgence of tobacco must, if injurious, be capable of abundant evidence, and this has never been fairly or fully shown. The mere fact of a man dying who used tobacco, or of a development of cancerous growth in the mouth, prove as well, in the first instance, that death ensued because a sufficient quantity was not used, and in the second place that an hereditary cachexia had been developed by an incident not at all connected with the cause. This wide spread instinct must have solid and undeniable proof to check its wonderful increase, not its mere denouncement, for there is a singular inclination to believe in the beneficence of some luxuries and pleasures the temperate enjoyment of which man so eagerly, and we say, so harmlessly finds comfort in.

The only fact established worthy of consideration, is the waste of the secretions of the mouth, which is said to produce derangement of the digestion. That a large amount of the saliva is thus wasted and if no compensation is made by the system for this loss, injury must inevitably result, but that indigestion follows as a necessary consequence is by no means proven, for the most frequent sufferers from indigestion are women who never use tobacco, nor has our observation established the fact that tobacco users are more liable to this malady than others.

We are compelled to think that the system compensates for this waste of the saliva by increasing the amount of the secretion, experiment establishing the fact that its quantity does not deteriorate the quality, if one pound and a half is the common quantity secreted, three times this weight can be produced without deranging its office in the stomach, a quantity quite sufficient to supply the wants of the system, and the most abandoned use of tobacco.

Physiology determines the use of this secretion to be two-fold, first, the moistening of food, and secondly, the conversion of farinaceous substances into sugar, and as this is so essential a feature in digestion, nature has supplied a reserve force in the *pancreas*, which has been determined by Kölliker and others to secrete an exactly similar fluid which is discharged upon the food just after it leaves the stomach, so that if any starchy matters have failed to become sufficiently saccharine for suitable appropriation, this gland supplies the want, and like all other glands is capable of increasing its usual supply to a sufficient extent.

Food is the natural stimulant of the salivary secretions, they are also excited by any mechanical substance placed in the mouth, tobacco is however the direct stimulant of these glands, and must be regarded not as the source of injury in its use, for the power of the system to fully supply an almost unlimited demand, is abundantly established by experiment. An apparent exhaustion by any artificial means will be at once corrected by a resort to the natural excitant, food, of almost any savory kind, showing that nature has power to reserve this secretion for natural demands. The injurious effects of tobacco must be found to attack some other function than that of the salivary secretion, and this has been supposed to be in its action upon the nervous system.

This vague and indefinite charge like the others against the use of this plant, is quite out of the domain of positive proof. Reasoning by analogies however, we are induced to draw rather a favorable hypothesis of its action upon the system, not forgetting that there are many idiosyncrasies quite incompatible with any habit out of the ordinary economy of nature.

Tobacco acts generally as an excitant and is an active stimulant to the nerves of sensation and emotion, thus its pleasurable effects are derived. It stimulates the brain proper, and until habit has modified its action, is followed by a depressing influence, which eventually becomes by continued use of a sedative character, lulling and soothing to the individual. It certainly has no direct action upon the sympathetic system of nerves, except when swallowed or used medicinally, and then it acts as a powerful revulsive.

As usual its action must be promotive of bodily comfort, reasoning from the laws of general health. In digestion its consequences must be, if any, in the stomach, by depriving it of the salivary secretion of the mouth, whilst the food is in this cavity, but when once the aliment has entered the stomach the availability of the secretion is prohibited by dilution and absence of contact. Digestion must proceed according to the powers of the stomach, and suitable appropriation take place according to the perfection of this process and the assimilating power of the system. Now all influences disturbing these processes act injuriously by diverting the concentration of vital action from the stomach and assimilative organs, and that which will remove any obstruction to digestion must necessarily allow a fuller action by removing impediments more or less great. Tobacco, therefore, in its sedative and narcotic influence upon the emotions and brain proper, relieves digestion of diverting influences, having similar tendencies as sleep, which allows all the vital force of the body to concentrate itself in the great work of digestion.

Opium acts very differently by first narcotising and then irritating, acting as well upon the sympathetic nerve as upon the brain, exhausting and paralyzing in its ultimate effect.

Too many are accustomed to class the two as similar evils, attributing all sorts of morbid consequences alike to their use. The brain under opium is not soothed or lulled into rest, it is simply benumbed, partially paralyzed, and has often a fearful reaction, deranges the secretions by suspending some and over stimulating others, operating through the nervous system upon the whole animal economy, whilst tobacco is incapable of any of these, exciting at first, but generally soothing and quieting in its permanent effect. In far the majority of persons that tobacco may be said to injure, there will be found a constitutional debility with habits and occupations, increasing this natural tendency, and thus inherited and acquired evils are mostly charged upon tobacco, when at worst it may have only added to an unavoidable decay, for these same conditions are as frequently found in persons who do not use it in any manner, as in those who do. Indeed we venture little in asserting that among women, who cannot be said to use tobacco, that there will be found as much impaired nutrition, more indigestion and derangement of the various secretions, of the nervous system, and especially of the liver, than can be found among chewers or smokers. Of course this is a mere assertion, but is it not the proverbial observation, and does not the practice of medicine indubitably point to woman as those most frequently demanding the aid of the physician?

It must be borne in mind that we are not advocating that tobacco is in all cases harmless, for we admit that its excessive use is always more or less injurious, productive of minor evils, principally perhaps of the nervous system, but claim that it seldom runs into thc course of grave disease, as no well authenticated case of death has been proven attributable to it. Its moderate use by individuals of no particular proclivity to disease, must be recognized as inoffensive, but to invalids and those of peculiar idiosyncrasies all habits are injurious that produce any disturbance, except those of a curative character. Prof. Christison affirms this position by saying, "no well ascertained ill effects have been shown to result from the habitual practice of smoking," much the most questionable form in which tobacco can be used. Beck states that "common observation settles the question, that the moderate and daily use of tobacco does not prove injurious, and this is the general rule," exceptions necessarily exist. Critical inquiry will establish beyond cavil, that the use of the plant is much less injurious than all other narcotics, opium, Indian hemp, the betel and the hop, have all distinct and positive evils in their train,* whilst tobacco has had simply vague charges of serious disease without proof or substantiation.

We are not forgetting that the Turk is perhaps the most abandoned user, as well as the most retrograding of civilized man, but all enlightened judgment will trace this to more radical causes than this simple habit. His geographical position, climate, religion, social and political government, all proclaim him to be the victim of destiny, and the progenitor of increasing moral and physical infirmity.

That tobacco is preservative of the teeth is by far the easiest of proof. The teeth decay principally from long contact with matters found in the mouth, whether it be acids, alkalies, or putrescent substances: it requires the action of one or all of these for a continued length of time to produce decay, so much so, that this would fail to produce this effect, did not the mechanical form of the teeth aid this result through the indentations, fractures and fissures in the enamel, admitting these destructive agents into the interior of the tooth, where the operation of exchange of elements takes place by a slow but unobstructed process.

Often the health of the mouth has really little to do with the decay of the teeth, for the gums and the various secretions may have been perfectly healthy, but from the form of the teeth admitting fluids to come in contact with the dentine, and there remaining until their destructive char-

* Christison reports the observations of MM. Parent, Duchatelet and D'Arcet, "among four thousand workmen in the tobacco manufactories of France, that they found no evidence of its being unwholesome."

acter is completed, when decomposition of the bone must go on until these causes are removed.

The greatest source of caries in the teeth, however, is found in the unhealthy condition of the fluids of the mouth and the want of absolute cleanliness, in order that these corroding agents may be entirely removed, and any procedure which will accomplish this end will effectually avoid the consequences with the previous exception considered. We believe the most visionary anti-nicotianist has never supposed that tobacco diseased the glands of the mouth, and we have shown that the quality of the salivary secretions is not altered in the least, being confined exactly to quantity. We will not stop, therefore, to predicate any hypothesis of disease, but simply claim for its use the preservation from decay in the teeth, from the frequent removal of the secretions, thus preventing their lodgment and consequent alteration or decomposition. Not only as to the fluids themselves, but particles of food which become loosened are ejected from the mouth which otherwise would remain and decompose. In chewing, a double purpose is fulfilled by adding to the foregoing the mechanical friction of the tobacco itself, which induces cleanliness as well as health to the gums, by hardening their surfaces and giving as it were a kind of exercise or action promotive of health.

In the elements of tobacco, there is nothing which can possibly attack the substance of the teeth, the organic acids of the plant are too mild and must be inoperative, the organic bases as lime, potassa, ammonia, &c., can only act as antagonistic to destructive acids, whilst the nicotin is protective of all animal tissues from decomposition, so that here is positive good resulting.

That it can have a reflective bad effect upon the teeth, is impossible to suppose, from the entire absence of any proof of its being the cause of any grave disease or organic lesion which alone induces a secondary action. Observation will more clearly establish this. The best natural dentures coming under the writer's notice has been in the mouths of tobacco chewers of middle aged men, whilst women have proverbially much less perfect teeth than man, and we may fairly claim that part of this greater perfection may be derived from the habits of using tobacco, and we therefore claim that it has rather a kindly and preservative influence upon the teeth. B.

---

### ARTICLE II.

### *Treatment of Sensitive Dentine.*

